# High-grade endometrial stromal sarcoma as the initial presentation of an adult patient with Peutz-Jeghers Syndrome: a case report

**DOI:** 10.1186/s13053-015-0027-0

**Published:** 2015-01-23

**Authors:** Maria Fernanda Noriega-Iriondo, Gerardo Colon-Otero, Benjamin R Kipp, John A Copland, Matthew J Ferber, Laura A Marlow, Maegan E Roberts, Matthew W Robertson, Tri A Dinh, Steven Attia, Xochiquetzal J Geiger, Douglas L Riegert-Johnson

**Affiliations:** 1 Centro Universitario contra el Cáncer, Av. Madero U Gonzalitos S/N, Monterrey, Nuevo León Mexico; 2 Division of Hematology/Oncology, Mayo Clinic, 4500 San Pablo Road, Jacksonville, 32224 FL USA; 3 Department of Surgical Gynecology, Mayo Clinic, 4500 San Pablo Road, Jacksonville, 32224 FL USA; 4 Department of Laboratory Medicine and Pathology, Mayo Clinic, 4500 San Pablo Road, Jacksonville, 32224 FL USA; 5 Department of Gastroenterology and Hepatology, Mayo Clinic, 4500 San Pablo Road, Jacksonville, 32224 FL USA; 6 Department of Cancer Cell Biology, Mayo Clinic, 4500 San Pablo Road, Jacksonville, 32224 FL USA; 7 Department of Laboratory Medicine, Mayo Clinic, Rochester, 55902 MN USA

**Keywords:** Peutz-Jeghers syndrome, Endometrial stromal sarcomas, Soft tissue sarcomas, Gynecologic oncology, Everolimus

## Abstract

A 46-year-old female presents with a pelvic mass and is diagnosed as having a high-grade endometrial stromal sarcoma. During surgery, she is noted to have areas of intussusception of the small bowel secondary to large hamartomatous polyps. The patient had a previous history of small bowel obstruction secondary to what had been thought to be hyperplastic polyps but represented hamartomatous polyps on further review. Additional examination revealed the presence of subtle hyperpigmented macules on the fingers leading to a diagnosis of Peutz-Jeghers Syndrome (PJS). The diagnosis was confirmed by the presence of a germ-line *STK11* mutation. Immunohistochemistry analysis of the tumor showed decreased expression of STK-11 as compared to one of the patient’s hamartomatous polyps. Next generation sequencing of the tumor specimen failed to demonstrate a “second hit” somatic mutation in *STK*-*11*. This case represents the first case of endometrial stromal sarcoma associated with PJS and illustrates the importance of increased awareness of this condition among oncologists. PJS is associated with dysregulation of the mTOR pathway; treatment with an mTOR inhibitor was not effective in this case.

**Electronic supplementary material:**

The online version of this article (doi:10.1186/s13053-015-0027-0) contains supplementary material, which is available to authorized users.

## Background

Peutz-Jeghers syndrome (PJS) is a rare, inherited disease, with an estimated incidence of one in 100,000. It is an autosomal dominant condition characterized by the development of hamartomatous polyps throughout the gastrointestinal tract (most commonly seen in the small bowel, particularly the jejunum), characteristic mucocutaneous pigmented lesions and elevated cancer risk [1]-[3].

Polyps may cause gastrointestinal bleeding, intussusception, obstruction, or infarction [1],[4]. The melanotic or lentiginous pigmented macules are usually located on the vermilion border of the lips, buccal mucosa, digits, and less frequently on the periorbital, auricular, perianal and vulvar skin [2]. Most patients are diagnosed early in life when they present with polyp-related complications. Malignant transformation of the polyps is rare.

This genetic condition is caused by a germline mutation in the tumor suppressor gene serine threonine kinase 11 gene *(STK11*, also known as *LKB1*) [5]. It is thought that all patients with PJS have a deleterious mutation in *STK11*, but current technology detects a mutation in only 75% of cases. The diagnosis does not usually require genetic confirmation; it can be made based on clinical presentation. Diagnostic criteria are outlined in Table 1[6].

**Table 1:** Diagnostic criteria for Peutz Jeghers Syndrome (PJS)

A. In patients without a family history of PJS, one of the following must be present:	• Characteristic melanotic macules and one or more intestinal polyps with PJS-type histology*, or
	• Two intestinal polyps with PJS-type histology*
B. In patients with a family history of PJS in a first degree relative, one of the following must be present:	• Characteristic melanotic macules, or
	• One intestinal polyp with PJS-type histology*, or
	• *STK11* mutation

Although the mechanism of carcinogenesis remains debatable, PJS patients carry an increased risk for the development of cancers of the gastrointestinal tract (colon, stomach, small intestine, and pancreas) as well as of non-gastrointestinal origin (breast and gynecological tumors) [4],[7].

PJS has not been associated with soft tissue sarcomas. In this article, we report the first case of a Peutz-Jeghers Syndrome patient with an endometrial stromal sarcoma.

## Case presentation

A 46-year-old woman presented with sensation of pressure in her pelvis. A CT scan of the abdomen and pelvis showed an 8 × 7 cm pelvic mass. Serum CA 125 level was 280 U/ml and serum CEA value was within normal limits. The patient underwent an exploratory laparotomy with findings of an 8 cm pelvic mass thought to be associated with an area of endometriosis with involvement of the pelvic *cul-de-sac* and pelvic retroperitoneum. Pathology was most consistent with a high grade endometrial stromal sarcoma. Adequate surgical cytoreduction and staging with hysterectomy, bilateral salpingo-oophorectomy, omentectomy, nodal sampling, and peritoneal washings were performed. No additional areas of tumor involvement were noted; specifically, there was no gross evidence of malignancy involving the uterus, fallopian tubes, or ovaries.

**Figure 1:**
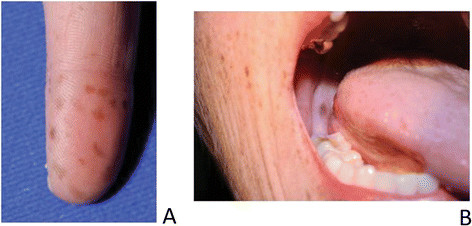
**Physical findings in the patient. A)** Multiple melanotic macules on the fingertips characteristic or Peutz-Jeghers syndrome, **B)** Melanotic macule on the right cheek.

During inspection of the small intestine, an area of intussusception was noted, with resection revealing the presence of two large pedunculated polyps (3.2 and 3.0 cm) as the cause of the intussusception. Suspicion of PJS was raised by intraoperative consultation with medical genetics based on the presence of pedunculated small bowel polyps and additional findings on physical examination of the patient, which included multiple hyperpigmented areas on her fingertips (Figure 1A).

**Figure 2:**
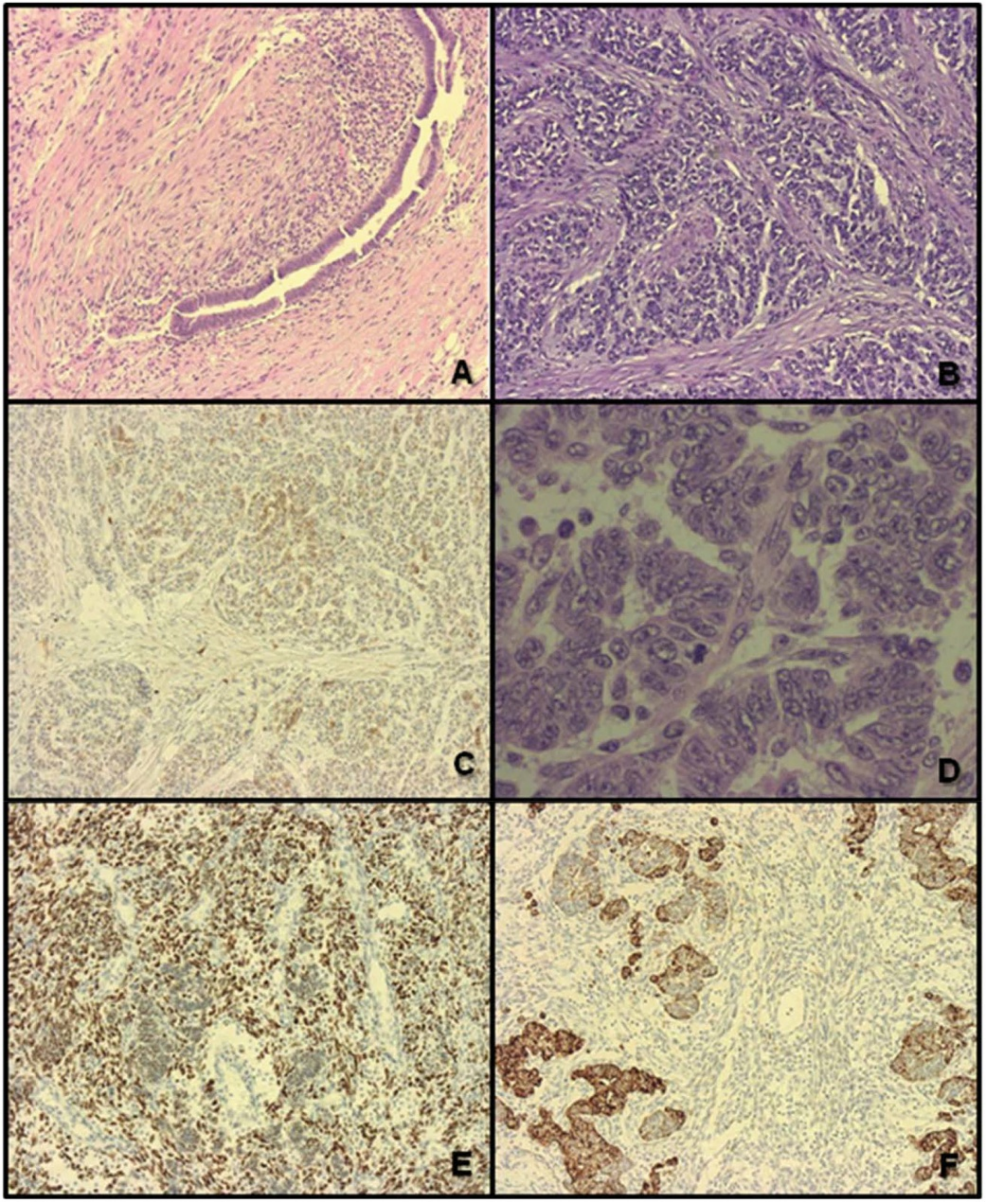
**Pathologic findings of the patient’s tumor. A)** Area of endometriosis adjacent to the tumor. **B)** Endometrial stromal sarcoma, low grade. **C)** Endometrial stromal sarcoma, low grade area, focally positive for CD10. **D)** Endometrial stromal sarcoma, epithelioid component. **E)** Endometrial stromal sarcoma spindle cell component estrogen receptor positive **F)** Endometrial stromal sarcoma, epithelioid component, Pancytokeratin positive.

Pathological examination showed a 9.5 cm malignant neoplasm consistent with high-grade endometrioid stromal sarcoma with a predominant epithelioid component (Figure 2A-F) and a definitive sarcomatous component with prominent sex cord differentiation. An area of endometriosis associated with the tumor was noted, raising the possibility that this pelvic tumor originated from transformation of benign endometriosis tissue. The malignant cells were positive for estrogen receptor, progesterone receptor, inhibin and CD10, with the more epithelioid areas positive for pancytokeratin, calretinin, D2-40, and MOC-31. CK7, CK20, desmin, actin, S-100, EMA, CEA, C-kit and CK-5/6 were negative. Differential diagnosis included the diagnosis of Malignant Female Adnexal Tumor of Wolffian Origin (FATWO). Evidence against this diagnosis was the high-grade nature of this tumor, the spindle cell component, and the negative staining for CK7, positive staining for EMA and negative staining for c-kit. Ovaries and fallopian tubes were negative for malignancy. The uterus had proliferative endometrium and unremarkable myometrium. Microscopic focal subserosal uterine tumor implants and para-uterine soft tissue tumor implants were present. Pelvic and *cul de sac* peritoneum were involved by tumor, and foci of endometriosis were present. Lymph nodes were negative for neoplasm (9 right para-aortic, 3 right external iliac, 8 right obturator, 4 left para-aortic, 3 left external iliac, and 6 left obturator nodes). In addition to the two larger polyps, six other small bowel polyps (0.4 – 2.2 cm) were found and resected via small bowel enterotomies; pathology on all polyps was significant for hamartomatous polyps of Peutz-Jeghers type. A Peutz-Jeghers type polyp is a hamartomatous polyp with smooth muscle arborization with occasional pseudo-invasion. This patient had three polyps removed from the large bowel a month before presenting to our institution; all three polyps were considered to be hyperplastic by an outside pathologist. Upon review of these polyps at our institution, it was determined that all three polyps were hamartomatous with one consistent with the Peutz-Jeghers type. Full gene sequencing and deletion/duplication analysis of the *STK11* gene was completed so that predictive testing could be offered to the patient’s daughter; this genetic testing was not needed to make the diagnosis of PJS in our patient. The diagnosis of Peutz-Jeghers syndrome was further confirmed with the identification of a deleterious mutation in the *STK11* gene, designated as 5’UTR_EX1del.

The patient received radiation therapy to the pelvis followed by chemotherapy consisting of ifosfamide and doxorubicin for 4 cycles. A repeat CT scan at the end of chemotherapy showed subcapsular liver nodules suspicious for metastasis. The patient was then started on letrozole, with development of progressive disease on this treatment. She developed small bowel obstruction requiring surgery with findings of high-grade small bowel obstruction requiring small bowel resection and multiple metastatic nodules involving the right hemi-diaphragm largest 3 cm in size and the transverse colon mesentery, largest 5 mm in size. Pathology showed a poorly differentiated neoplasm with a predominant epithelioid component and a minimal spindle cell component. The epithelioid component was pankeratin positive, with patchy EMA positivity, patchy CK8/18 positivity and patchy membranous CD99 positivity. Tumor cells were ER positive (60%) and PR positive (5-10%) and were negative for inhibin, melan A 103, desmin and SMA. The spindle cell component had a myxoid morphology with patchy faint positivity for SMMS-1. Subsequent treatment with everolimus and anastrozole and later on with pazopanib and everolimus, and gemcitabine and docetaxel, was not associated with a clinical response. The patient had progressive disease leading to her death.

Additional genetic and immunohistochemical (IHC) analysis of the tumor specimens were performed. Tumor DNA was extracted from formalin fixed paraffin embedded tissue. STK11 was sequenced using custom sequence capture with targeted next generation sequencing. IHC of the hamartomatous polyps and the sarcoma tumor from paraffin embedded tissue was done utilizing human LKB1/STK11 Affinity Purified Polyclonal Antibody (R & D Systems).

**Figure 3:**
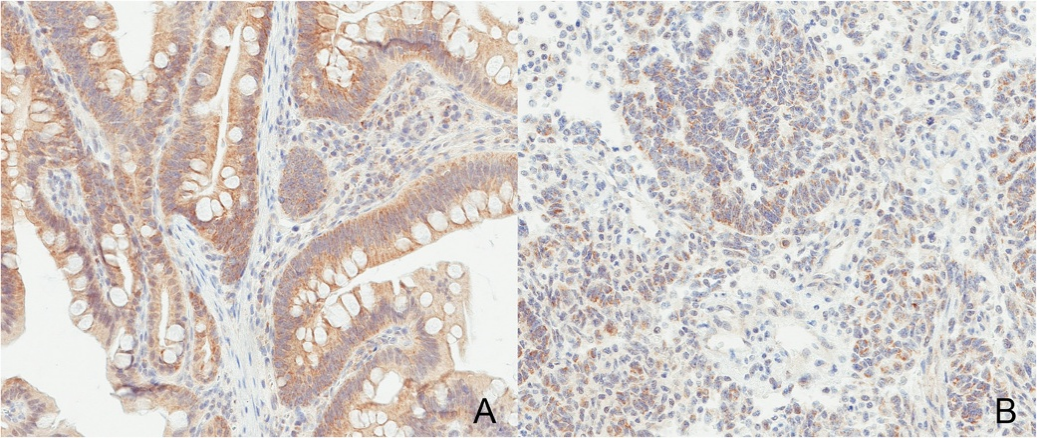
**Immunohistochemistry (IHC) for STK-11 of patient’s tissues. A)** IHC for STK-11 of the hamartomatous polyp and **B)** the endometrial stromal sarcoma. Images were obtained at 20X using Scanscope XT (Aperio Technologies, Vista, CA).

STK11 tumor sequencing findings were consistent with the patient's known germline deletion of exon 1. A "second hit" somatic tumor mutation was not identified. IHC studies demonstrated STK-11 expression in the hamartomatous polyp with decreased expression of STK-11 in the sarcoma tumor tissue (Figure 3).

## Conclusions

PJS patients have a higher risk for malignancies at a young age, especially colorectal, breast, gynecologic, small bowel, gastric, and pancreatic cancer. The relative cancer risk by site is estimated to vary between 4.8 to 18 times that of the general population, with a lifetime cumulative cancer risk up to 93% [7]. The risk is much higher in female patients than male patients since gynecologic malignancies and breast cancers are two of the most common malignancies associated with PJS. Among the gynecologic malignancies, adenoma malignum of the uterine cervix, adenocarcinoma of the endometrium and ovarian tumors have been estimated to have a 15-fold higher incidence than the general population [8].

Clinical suspicion for PJS needs to remain high in order to make the diagnosis. The hyperpigmented melanotic lesions may be missed since they may be few in number, fade with age, and present most prominently on the fingers and in the oral cavity without being noticeable on the lips as in our patient (Figure 1A-B). The hamartomatous polyps maybe misclassified as hyperplastic polyps and therefore the connection between the gynecological malignancy, the polyps and hyperpigmentation may escape the diagnosis of PJS until adulthood as occurred in our patient.

The most common ovarian neoplasm is the sex cords tumor with annular tubules (SCTAT), which is typically multifocal, calcified, and bilateral. Approximately 10% of patients with PJS will develop SCTATs that require surgery. These tumors are usually of a low malignant potential and carry a good prognosis [6],[9].

Adenoma malignum (ADM) is a rare variant type of highly differentiated adenocarcinoma of the endocervical glands that comprises 1-3% of all cervical adenocarcinomas; it can be aggressive and have a poor prognosis despite its benign histologic appearance. The percentage of patients with PJS who develop ADM is 5% or less, and about 10% of patients with ADM have PJS [8],[10].

Sarcomas are not usually associated with PJS. There is only one case report of an epithelioid leiomyosarcoma originating in a small bowel hamartomatous polyp in a patient with PJS. The tumor showed an aggressive behavior and by the time of resection, it had metastasized to the liver. This is very unusual since malignant transformation of the hamartomatous polyps is rare [11]. There are no reports of gynecological or pelvic sarcomas or of endometrial stromal sarcomas in patients with PJS.

We attempted to identify a "second hit" somatic tumor mutation to conclusively link the tumor to the patient's PJS diagnosis and germline *STK11* mutation. The lack of a "second hit" does not disprove that the patient's tumor was related to her PJS diagnosis. "Second hit" somatic mutations are only found in about 1 in 5 samples from PJS intestinal polyps [12]. Explanations for the lack of identification of a "second hit" include promoter hypermethylation, intronic mutations and complex rearrangements. The relative decrease in the expression of the STK11 protein in the sarcoma tumor compared to the hamartomatous polyp as determined by IHC does support a link between the tumor and the patient’s PJS diagnosis.

Identification of *STK11* gene mutations in PJS have allowed for the development of targeted molecular therapy. *STK11* mutations are associated with activation of the mTOR pathway. mTOR inhibitors have been developed for clinical use in organ transplantation and treatment of renal cancers. The mTOR inhibitors have been reported in the treatment of a PJS patient with pancreatic cancer and could possibly serve as a second line treatment for patients who have failed to respond to other therapies [13]. The promise of these targeted therapies for this rare cancer is another important reason for oncologists to be more aware of PJS and its potential diagnostic challenges. Unfortunately, the patient’s tumor did not respond to mTOR inhibition.

## Consent

Written conformed consent was obtained from the patient for publication of this case report and any accompanying images. A copy of the written consent is available for review by the Editor in Chief of this journal.

## Authors’ original submitted files for images

Below are the links to the authors’ original submitted files for images. Authors’ original file for figure 1 Authors’ original file for figure 2 Authors’ original file for figure 3

## Abbreviations

ADM: Adenoma malignum
CT: Computed tomography
PJS: Peutz-Jeghers Syndrome
SCTAT: Sex cords tumor with annular tubules
