# Supplementary figures and images for: High-grade endometrial stromal sarcoma as the initial presentation of an adult patient with Peutz-Jeghers Syndrome: a case report
Source: Hered Cancer Clin Pract. 2015 Jan 23;13:6. doi: 10.1186/s13053-015-0027-0 (PMC4314827; doi:10.1186/s13053-015-0027-0)

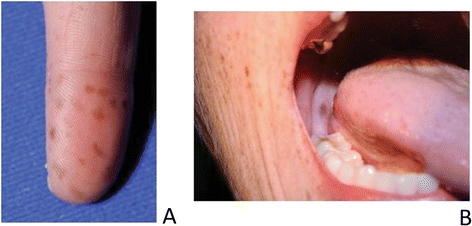

Supplement: Supplementary file 1 — Authors’ original file for figure 1 [file 13053_2015_27_MOESM1_ESM.gif]

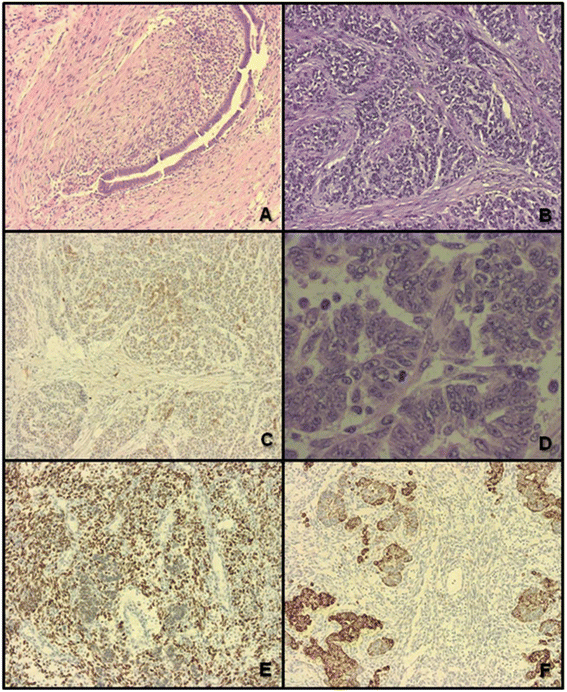

Supplement: Supplementary file 2 — Authors’ original file for figure 2 [file 13053_2015_27_MOESM2_ESM.gif]

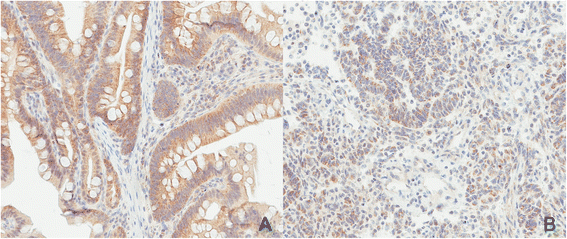

Supplement: Supplementary file 3 — Authors’ original file for figure 3 [file 13053_2015_27_MOESM3_ESM.gif]
